# GH successful treatment in a female with a de novo 46,XX,add(X)(p36),t(X;Y)(p36.3;p11.2), growth impairment and SHOX-haploinsufficiency

**DOI:** 10.1186/s13052-019-0694-y

**Published:** 2019-08-14

**Authors:** Maria Cristina Maggio, Giovanni Corsello

**Affiliations:** Department of Health Promotion Sciences Maternal and Infantile Care, Internal Medicine and Medical Specialities “G. D’Alessandro”, University of Palermo, Palermo, Italy

**Keywords:** SHOX haploinsufficiency, Growth hormone, Treatment adherence, Chromosome translocation

## Abstract

Children with chromosome translocations, concerning X chromosome, have a genetic pattern different from Turner syndrome; however, when a translocation involves the of part of X chromosome including short stature homeobox-containing Sex-determining Region Y gene, growth may be severely compromised.

We describe the clinical case of a 2.2-year-old-female, arrived at our paediatric unit for a decrease of height velocity. The karyotype was 46,XX,add(X)(p36.3). Array comparative genomic hybridization showed a fragment of Y chromosome, extended from 8.803.981 (Yp11.2) to 28.767.604 (Yq11.23). The final karyotype was 46,XX,add(X)(p36),t(X;Y)(p36.3;p11.2). Fluorescence in situ Hybridization analysis using Sex-determining Region Y probe revealed no signal on the derivative Y chromosome.

At the admittance, height was 84.5 cm (− 1.24 SDS); SPAN was 79 cm; sitting height: 72.4 cm; weight was 17.5 kg. Bone age was 1.2 years. Multiplex Ligation Probe Amplification showed a heterozygous deletion of the Short Stature Homeobox-containing gene and of the pseudoautosomal region-1. This result correlated with Leri-Weill Syndrome. She started Growth Hormone treatment, with a good response.

The case described shows a rare translocation, involving the X chromosome and including SHOX gene and the pseudoautosomal region-1. At our knowledge, this is the first case of a patient with a karyotype 46,XX,add(X)(p36),t(X;Y)(p36.3;p11.2) and Short Stature Homeobox-containing gene haploinsufficiency, successfully treated with Growth Hormone.

Dear Sir,

Children with chromosome translocations, concerning X chromosome, could have growth deficiency. This condition is different from Turner syndrome, when both X chromosomes are present and do not include deletions. However, when a translocation involves the of part of X chromosome including SHOX gene, growth may be severely compromised [1, 2].

We describe the clinical case of a 2.2-year-old-female, arrived at our paediatric unit for a decrease of height velocity.

She was born Small for Gestational Age (SGA) at 38.6 weeks; at birth, weight was 2710 g (− 1.05 SDS; 15° Centile); length was 44 cm (− 2.45 SDS; 1°Centile); cranial circumference was 33.5 cm (− 0.19 SDS; 42° Centile). She showed dysmorphic traits (single transverse palmar crease) and a karyotype was evaluated, to exclude anomalies. The karyotype was 46,XX,add(X)(p36.3). A further evaluation by array comparative genomic hybridization (CGH) showed a fragment of Y chromosome, extended from 8.803.981 (Yp11.2) to 28.767.604 (Yq11.23). The final karyotype was 46,XX,add(X)(p36),t(X;Y)(p36.3;p11.2). This fragment of Y chromosome did not include Sex-determining Region Y gene (SRY) gene. In fact, Fluorescence in situ Hybridization (FISH) technique using SRY probe revealed no signal on the derivative Y chromosome in this patient.

At the admittance, height was 84.5 cm (− 1.34 SDS, according to the SIEDP growth charts performed for south Italy and islands [3]); SPAN was 79 cm; sitting height: 52.4 cm; weight was 17.5 kg (2.06 SDS); bone age was 1.2 years.

The father height is: 168 cm; the mother height: 150 cm, with a genetic target of 152.5 cm (− 1.65 SDS, according to the SIEDP growth charts performed for south Italy and islands).

She showed short forearm, cubitus valgus, suggesting Short Stature Homeobox-containing gene (SHOX) haploinsufficiency [2].

The genetic study of SHOX gene was performed in multiplex ligation-dependent probe amplification (MLPA) and showed a heterozygous deletion of the region L10292 (hg18loc.X-000,227,417) - L25227 (hg18loc.X-002,418,479) for a trait of 2191 kb. The deletion includes SHOX gene and PAR1 region. This result correlated with Leri-Weill Syndrome.

She started Growth Hormone (GH) treatment at the dosage of 0.03 mg/kg/day [4, 5], with a treatment adherence of 98%, in part permitted by the recent beginning of GH treatment. Recently, the treatment adherence was inversely related to patients’ age and showed an inverse correlation with the years of the therapy [6]. The increase of growth was associated with increased IGF-1 levels, whose levels, however, were maintained in the normal range. At 3.7 years, the height was 95.5 cm (− 1.11 SDS); the weight was 17.5 kg (0.57 SDS); SPAN was 88.5 cm. At 5.1 years, the height was 106.5 cm (− 0.81 SDS); the SPAN was 102 cm; sitting height: 92.9 cm; the weight was 22.5 kg (0.78 SDS); bone age was: 4 years and the radiogram of the forearm documented an initial Madelung deformity, with shortening and bowing of the radius.

Pelvic ultrasound study, beta-human chorionic gonadotropin, alpha-fetoprotein, evaluated at the diagnosis and during the follow up, were in the normal range.

Translocations involving the human sex chromosomes are rarely reported and regulate the phenotype, in relation with the presence of SRY in the Y chromosome. The effects of such traslocations are disorder of sex development (DSD), 46, XX DSD, and 46,XY DSD. In some rare cases of abnormal sex determination, a complete sex reversal is reported, with XY females or XX males. It is determining the exchange in the paternal germ line of terminal portions of Yp 11.2 (cytognetic location of SRY) and Xp or other autosomes [7].

In fact, the presence of the SRY gene in the normal male zygote leads to the development of male genital organs and the absence of this gene leads to the development of a female phenotype.

In our patient the translocation did not include SRY region, explaining the absence of masculinization, with a normal female phenotype of internal and external genitalia.

The case described however, shows a rare translocation, involving the X chromosome and including SHOX gene and PAR-1 region. However, the phenotype of Leri Weil Syndrome suggested to perform the genetic study of SHOX gene, demonstrating a wide gene deletion.

At our knowledge, this is the first case of a patient with a karyotype 46,XX,add(X)(p36),t(X;Y)(p36.3;p11.2) and SHOX-haploinsufficiency, successfully treated with GH.

The treatment was not suggested as “Turner Syndrome”, because the karyotype was not confirmatory of this condition. Conversely, SHOX haploinsufficiency allowed to start precociously GH treatment with an improvement in growth velocity and an increase in SDS for stature of 0.53 SDS. In fact, early treatment in SHOX haploinsufficiency contribute to ameliorate adult height [5]. The treatment is useful also if height velocity is decreased at the beginning of treatment, although stature is not still under − 2 SDS, as relieved in our patient.

## Data Availability

Materials and data of the patient are included in the medical records of the patient.

## Abbreviations

CGH: Comparative genomic hybridization
DSD: Disorders of sex development
FISH: Fluorescence in situ Hybridization
GH: Growth Hormone
MLPA: Multiplex Ligation Probe Amplification
SGA: Small for Gestational Age
SHOX: Short Stature Homeobox-containing gene
SRY: Sex-determining Region Y gene
